# Supplementary material for: Evaluation of Microbial Dynamics of Kombucha Consortia upon Continuous Backslopping in Coffee and Orange Juice
Source: Foods. 2023 Sep 24;12(19):3545. doi: 10.3390/foods12193545 (PMC10572523; doi:10.3390/foods12193545)
Supplement: Supplementary file 1 [file foods-12-03545-s001.zip › Supplementary_materials_final.pdf]

Table S1 pH, TA and sensory characteristics of the initial kombucha in black tea.

| Day | pH | TA | Description of sensory |
|---|---|---|---|
| **0** | 4.3 | 0.0 | Taste like sweet black tea |
| **1** | 4.2 | 0.4 | Dominate sweet taste, some sour notes |
| **2** | 4.0 | 0.4 | Slightly acetic taste |
| **3** | 3.7 | 0.6 | Strongly carbonated, smells like apricot, some bitter notes |
| **4** | 3.6 | 0.7 | Strongly carbonated, good sweet-sour balance, acetic |
| **5** | 3.5 | 1.1 | Strongly carbonated, had over-ripened food taste |
| **6** | 3.4 | 1.8 | Strongly acetic and sour tase |
| **7** | 3.3 | 2.7 | Strongly carbonated, sour and acetic |
| **8** | 3.3 | 2.7 | Astringent and very acetic |

Table S2 pH, TA and sensory characteristics of the LAB-tailored kombucha in black tea.

| Day | pH | TA | Description of sensory |
|---|---|---|---|
| **0** | 4.3 | 0.0 | Taste like sweet black tea |
| **1** | 4.2 | 1.1 | Dominate sweet taste |
| **2** | 3.8 | 1.5 | Taste like peach ice tea, sweet |
| **3** | 3.6 | 2.0 | Taste like peach ice tea, sweet and a bit sour |
| **4** | 3.5 | 2.1 | Good sweet-sour balance |
| **5** | 3.4 | 2.6 | Carbonated, good sweet-sour balance |
| **6** | 3.3 | 3.0 | Carbonated, good sweet-sour balance, no acidic taste occurred |
| **7** | 3.2 | 3.4 | Dominate sour taste, the odour was characteristic to kombucha |
| **8** | 3.1 | 4.2 | Dominate strong sour taste |

Table S3 The lactic acid bacteria species abundance was measured by qPCR in LAB-tailored kombucha.

| Sampling point | Time (months) | ***Levilactobacillus brevis*, cells/mL** | ***Lactiplantibacillus plantarum*, cells/mL** | ***Pediococcus pentosaceus*, cells/mL** |
|---|---|---|---|---|
| I | 1 | 35000 | 230000 | 200000 |
| II | 2 | 2000 | 650 | 300 |
| III | 2.5 | 6 | 1 | 50 |
| IV | 3 | 70 | 80 | 5 |
| V | 4 | 100 | 10 | 60 |

Table S4 pH, TA and sensory characteristics of the orange juice kombucha in the prelimenary optimisation study.

| Day | pH | TA | Description of sensory |
|---|---|---|---|
| **0** | 4.2 | 5.0 | Taste like orange juice |
| **1** | 4.2 | 5.6 | Taste like orange juice |
| **2** | 4.1 | 6.4 | Orange juice, slightly sour and carbonated |
| **3** | 4.0 | 7.7 | Strongly carbonated, dominative sour taste |
| **4** | 3.9 | 9.1 | Strongly carbonated, chemical taste, dominative sour taste, astringent |

Table S5 Description of sensory characteristics during the orange juice kombucha backslopping (BS). The sensory was conducted by three assessors.

| Sample | Description of sensory |
|---|---|
| **BS 0.1** | Taste like orange juice |
| **BS 0.2** | Orange juice, slightly sour and carbonated |
| **BS 1.1** | Taste like orange juice |
| **BS 1.2** | Strongly carbonated, dominative sour taste |
| **BS 2.1** | Strongly carbonated, good sweet-sour balance |
| **BS 2.2** | Strongly carbonated, chemical taste, dominative sour taste |
| **BS 3.1** | Strongly carbonated, good sweet-sour balance |
| **BS 3.2** | Strongly carbonated, chemical taste, dominative sour taste, astringent |
| **BS 4.1** | Strongly carbonated, dominative sour taste |
| **BS 4.2** | Strongly carbonated, chemical taste, strong sour, and astringent taste |
| **BS 5.1** | Strongly carbonated, dominative sour taste |
| **BS 5.2** | Strongly carbonated, chemical taste, strong sour, and astringent taste |

Table S6 pH, TA and sensory characteristics of the coffeee kombucha in the prelimenary optimisation study

| Day | pH | TA | Description of sensory |
|---|---|---|---|
| **0** | - | - | Characteristic taste and odour of coffee |
| **1** | 4.6 | 0.9 | Characteristic taste and odour of coffee |
| **2** | 4.5 | 1.1 | Dominative taste of sweet coffee, more sweet than sour taste |
| **3** | 4.4 | 1.9 | Dominative taste of sweet coffee, balanced sweet and sour taste |
| **4** | 4.2 | 2.4 | Balanced sweet and sour taste, some notes of acetic acid |

| | | | |
|---|---|---|---|
| **5** | 4.2 | 3.2 | Dominative sour and bitter taste |

Table S7 Description of sensory characteristics during the coffee kombucha backslopping. The sensory was conducted by three assessors.

| Sample | Description of sensory |
|---|---|
| **BS 0.1** | Characteristic taste and odour of coffee |
| **BS 0.2** | Dominative taste of sweet coffee, balanced sweet and sour taste |
| **BS 0.3** | Dominative taste of sweet coffee, balanced sweet and sour taste |
| **BS 0.4** | Balanced sweet and sour taste, bitterness of coffee was stronger than a day before |
| **BS 1.1** | Characteristic taste and odour of coffee |
| **BS 1.2** | Dominative taste of sweet coffee, balanced sweet and sour taste |
| **BS 1.3** | Balanced sweet and sour taste, some notes of acetic acid |
| **BS 1.4** | Dominative sour and bitter taste |
| **BS 2.1** | Characteristic taste and odour of coffee |
| **BS 2.2** | Dominative taste of sweet coffee, balanced sweet and sour taste, slightly carbonated |
| **BS 2.3** | Dominative sour and bitter taste |
| **BS 2.4** | Dominative sour and acetic taste |
| **BS 3.1** | Characteristic taste and odour of coffee |
| **BS 3.2** | Dominative taste of sweet coffee, balanced sweet and sour taste, slightly carbonated |
| **BS 3.3** | Dominative taste of sweet coffee, balanced sweet and sour taste, slightly carbonated |
| **BS 3.4** | Dominative sour and very bitter taste, carbonated |
| **BS 4.1** | Characteristic taste and odour of coffee |
| **BS 4.2** | Dominative taste of sweet coffee, balanced sweet and sour taste, slightly carbonated |
| **BS 4.3** | Dominative sour taste, carbonated |
| **BS 4.4** | Intensively sour and bitter taste, carbonated |
| **BS 5.1** | Characteristic taste and odour of coffee |
| **BS 5.2** | Dominative taste of sweet coffee, balanced sweet and sour taste, slightly carbonated |
| **BS 5.3** | Dominative sour and bitter taste, carbonated |
| **BS 5.4** | Intensively sour and bitter taste, carbonated |

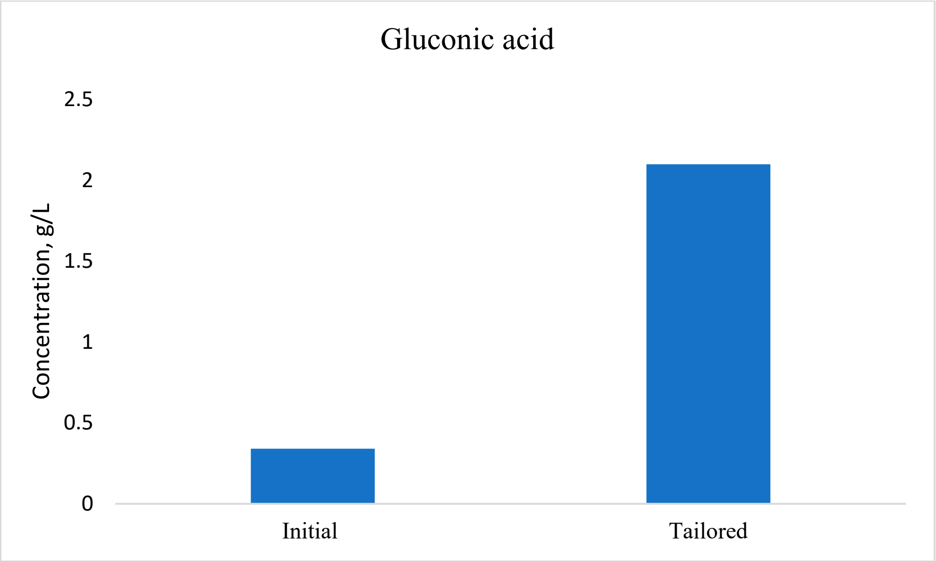

Figure S1 Gluconic acid concentrations in initial and LAB-tailored kombucha.
